# Comparison of Self-Reported and Accelerometer-Assessed Physical Activity in Older Women

**DOI:** 10.1371/journal.pone.0145950

**Published:** 2015-12-29

**Authors:** Eric J. Shiroma, Nancy R. Cook, JoAnn E. Manson, Julie E. Buring, Eric B. Rimm, I-Min Lee

**Affiliations:** 1 Division of Preventive Medicine, Brigham & Women’s Hospital, Harvard Medical School, Boston, MA, United States of America; 2 Department of Epidemiology, Harvard School of Public Health, Boston, MA, United States of America; 3 Department of Nutrition, Harvard School of Public Health, Boston, MA, United States of America; 4 Laboratory of Epidemiology and Population Science, Intramural Research Program of the National Institutes of Health, National Institute on Aging, Bethesda, MD, United States of America; Vanderbilt University, UNITED STATES

## Abstract

**Background:**

Self-reported physical activity measures continue to be validated against accelerometers; however, the absence of standardized, accelerometer moderate-to-vigorous physical activity (MVPA) definitions has made comparisons across studies difficult. Furthermore, recent accelerometer models assess accelerations in three axes, instead of only the vertical axis, but validation studies have yet to take incorporate triaxial data.

**Methods:**

Participants (n = 10 115) from the Women’s Health Study wore a hip-worn accelerometer (ActiGraph GT3X+) for seven days during waking hours (2011–2014). Women then completed a physical activity questionnaire. We compared self-reported with accelerometer-assessed MVPA, using four established cutpoints for MVPA: three using only vertical axis data (760, 1041 and 1952 counts per minute (cpm)) and one using triaxial data (2690 cpm).

**Results:**

According to self-reported physical activity, 66.6% of women met the US federal physical activity guidelines, engaging in ≥150 minutes per week of MVPA. The percent of women who met guidelines varied widely depending on the accelerometer MVPA definition (760 cpm: 50.0%, 1041 cpm: 33.0%, 1952 cpm: 13.4%, and 2690 cpm: 19.3%).

**Conclusions:**

Triaxial count data do not substantially reduce the difference between self-reported and accelerometer-assessed MVPA.

## Introduction

Physical activity has been consistently shown to be associated with reduced rates of cardiovascular disease, type 2 diabetes, certain cancers, and premature mortality in large observational studies.[1–4] [3, 5–8] Despite the multitude of health benefits associated with being physically active, a majority of Americans remain insufficiently active. Physical inactivity is reported to cause between 6% and 10% of major non-communicable diseases worldwide.[9] Accurate assessment of physical activity is necessary to inform recommendations and interventions to decrease physical inactivity.

Self-reported physical activity questionnaires remain the primary assessment method for large observational studies despite their limitations.[10, 11] Physical activity questionnaires rank participant physical activity levels moderately well, but are less precise assessing the absolute volume of physical activity (e.g. the total amount of time spent in moderate-to-vigorous physical activity (MVPA)) compared to objective measures.[12–15] Objective measures of physical activity, such as those obtained from accelerometers, may allow for a more precise assessment of physical activity volume.

It is important to understand the relationship between accelerometer-assessment and self-report. Accelerometers, due to their decreasing cost and size, have become increasingly prevalent in both research settings and as commercial products [16, 17], but are unlikely to fully replace self-report as the primary MVPA assessment method in large observational studies. Self-report questionnaires may be preferred due to fewer logistical challenges as well as to examine specific activities or domains of activity (such as leisure-time, transportation, occupational, and home-based physical activity).[17, 18] Finally, the majority of the existing research examining physical activity and health is based on self-reported physical activity.[3, 8]

Previous studies have shown a low to moderate correlation between self-report questionnaires and uniaxial accelerometer measures, as well as significant differences in absolute volume of MVPA measured.[18–26] A challenge to describing accelerometer-assessed physical activity is determining the appropriate cutpoint to translate accelerometer measures into physical activity carried out at different intensities. Evenson et al., in a nationally representative sample of adults aged 60 years and older, have shown different cutpoints influenced to a large extent the determination of time spent in MVPA.[27]

Numerous accelerometer cutpoints for MVPA, all using data collected from the vertical axis, have been proposed based on calibration studies primarily carried out under laboratory settings, but there is no consensus on the appropriate cutpoint,[28–32] particularly for older adults.[22, 27, 33] Several studies have demonstrated the need for the calibration of accelerometer cutpoints in an older population with a wide variety of activities conducted in a ‘free-living’ environment.[22, 27, 31] Since no ‘gold standard’ cutpoint for older adults exists, studies have used a variety of cutpoints to describe accelerometer-assessed time in MVPA. Furthermore, using data from all three axes of a triaxial accelerometer may provide a better assessment of activities being performed; however, no data currently are available on how self-reported physical activity compares with triaxial accelerometer measures.

Perhaps the largest challenge in comparing data collected using accelerometers or questionnaires lies in what each method truly measures. Accelerometers measure accelerations in physical motion, and do not directly measure behavior (e.g., 1 hour of tennis play does not involve 60 minutes of continuous movements).[16] While accelerometers offer the possibility of greater characterization of physical activity (e.g., identification of short bouts), innovative analytical methods such as pattern analysis, machine-learning, and the sedentary sphere hold promise, but are still under development.[34, 35] Given the increased use of accelerometers in research currently due to ease and the potential for deeper pattern analyses, its remains important to understand and quantify how physical activity assessed using questionnaires and accelerometers differ.

In a cohort of women aged 62 years and older, we compare accelerometer-assessed physical activity, using uniaxial and triaxial data and different accelerometer cutpoints, with self-reported physical activity.

## Methods

### Study participants

Participants were from the Women’s Health Study (WHS),[36–38] a randomized trial (clinicaltrials.gov: NCT00000479, 1992–2004) of aspirin and vitamin E for preventing cardiovascular disease and cancer among 39 876 healthy women aged >45 years. When the trial ended, 33 682 women (88.6% of those alive) consented to continue with observational follow-up, reporting on their health habits and medical history annually on questionnaires. An ancillary study was funded to examine accelerometer-measured physical activity and health outcomes; data collection began in 2011.

Women were mailed an accelerometer (ActiGraph GT3X+) and asked to wear it on the hip for 7 days during waking hours. They also completed a wear time diary, indicating which days the monitor was worn, and returned both the accelerometer and diary by mail. Women were then mailed a brief physical activity questionnaire (see below); inquiring about leisure time physical activity patterns during the time the monitor was worn. The study was approved by the institutional review board of Brigham and Women’s Hospital and women provided written consent to participate.

### Accelerometer-assessed physical activity

We defined accelerometer-assessed volume of physical activity and time in MVPA using four cutpoints to define activity of at least moderate-intensity. These were three widely applied cutpoints which use data from the vertical axis: 760 counts per minute (cpm),[31] 1041 cpm,[30] and 1952 cpm;[28] and the only triaxial (vector magnitude) cutpoint that has been proposed for adults: 2690 cpm [39]. Vector magnitude is calculated as the square root of the sum of squares of counts per minute from all three axes.

We calculated the number of minutes per week above each MVPA cutpoint, representing time spent in MVPA; and the average counts per day above each MVPA cutpoint, representing MVPA volume. Meeting physical activity guidelines was defined as ≥150 minutes per week where the accelerometer registered at least the MVPA cutpoint; not meeting, otherwise.[7] This definition was applied considering only MVPA occurring in bouts of ≥10 minutes’ duration as specified by the guidelines,[7] and separately, removing the bout length restriction, considering all MVPA including MVPA occurring in bouts shorter than 10 minutes’ duration.

### Self-reported physical activity

On the physical activity questionnaire examining the week the monitor was worn, women reported stairs climbed, usual walking pace, and time spent per week in 10 groups of leisure-time activities: walking or hiking; jogging; running; bicycling; aerobic exercise, such as aerobic dancing or exercise machines; lower-intensity exercise, such as yoga or stretching; tennis, squash, or racquetball; lap swimming; strength training; and other activities (S1 Fig).[11] For each activity or group of activities reported, we assigned a MET (metabolic equivalent) score, which represents the energy cost of the activity (e.g., resting quietly = 1 MET; walking briskly = 4 METs).[40] The physical activity volume, in MET-hours per week, for each activity or group of activities was calculated as a product of the time spent per week and the MET score. In a validity and reliability study, the questionnaire physical activity estimates yielded a correlation of 0.79 when compared to activity recalls and 0.62 when compared to activity diaries over 4 past-week assessments. The questionnaire test-retest correlation over 2 years was 0.59.[41]

We estimated the minutes of MVPA, a sum of the time spent during that week on reported activities ≥3 METs, as well as the MET-hours of MVPA, a sum of MET-hours across all reported activities ≥3 METs.[42] Meeting physical activity guidelines was defined as accumulating ≥150 minutes per week of moderate-intensity physical activity (≥3 METs and <6 METs), 75 minutes of vigorous-intensity physical activity (≥6 METs), or an equivalent combination.[7] While the current guidelines require activities to be performed in bouts of ≥10 minutes, we did not apply this restriction, as the physical activity questionnaire did not specify reporting of only activities that occurred in bouts of ≥10 minutes.

### Statistical analyses

We calculated the median number of minutes per week of MVPA by self-report and accelerometer-assessment based on the various cutpoints described above and the correlation of MVPA volume between self-report and accelerometer-assessment using the different cutpoints. Medians and Spearman partial correlation coefficients were reported, as the data were not normally distributed. The interquartile range (IQR) is represented by the 25^th^ and 75^th^ percentiles. Partial correlations were adjusted for age and accelerometer wear time.

To further describe the effect of the accelerometer MVPA cutpoints on the relationship with self-reported physical activity, we calculated the average counts per day and minutes of activity above vertical axis cutpoints ranging from 100 to 3900 cpm, in 200 cpm intervals (i.e., ≥100 cpm, ≥300 cpm, …, ≥3900 cpm). Then, we calculated the age and wear time adjusted correlation between accelerometer-assessed MVPA volume, based on each of these cutpoints, and self-reported MVPA volume to determine the cutpoint yielding the highest correlation. Additionally, we calculated the difference between time spent in MVPA assessed by accelerometer, using each of the vertical axis cutpoints, and self-reported MVPA to determine the cutpoint yielding the smallest difference. Parallel analyses were performed using vector magnitude cutpoints, over the range of 300 to 6000 cpm, in 300 cpm intervals.

We calculated the percent of women who met US federal physical activity guidelines [7] by self-report and accelerometer-assessment using the four cutpoints, described above that have been widely used in previous studies, to define activity of at least moderate-intensity. Parallel analyses were conducted restricting and not restricting the accelerometer-assessed physical activity to bouts of at least 10 minutes. To describe the classification accuracy, we calculated the kappa statistic, following the guidelines of Landis and Koch: kappa less than 0.40 indicates “poor” agreement, values between 0.40 and 0.75 indicate “moderate” agreement, and values greater than 0.75 indicate “excellent” agreement.[43]

## Results

As of 1 January 2014, 26 978 women had been invited to participate in the ancillary study and 23 934 (88.7%) responded. Of the 23 934, 1204 (5.0%) were ineligible because they were unable to walk independently outside the home (eligibility criterion). Among the remaining 22 730 women, 16 689 (73.4%) agreed to participate. As of 13 May 2014, 14 796 women had returned their accelerometers.

Accelerometer data, using all three axes, were screened for periods of wear (“wear time”) using methods described by Choi et al.[44] Briefly, non-wear time was defined as 90 consecutive minutes of zero counts, with an allowance of 2-minutes of nonzero counts provided there were 30-minute consecutive zero count windows up and downstream. Wear days were identified by the wear time diary. We excluded 245 women due to technical error with the accelerometer, 1850 women who did not return a diary, 2197 women who did not return a physical activity questionnaire, and 389 women who did not have ≥4 days of ≥10 hours of wear per day (convention for sufficient “valid data”), [45] resulting in 10 115 women.

Women who did not agree to participate in the ancillary study were similar in age (not willing: 71.0 vs valid data: 70.0), but had a higher body mass index (BMI) (not willing: 27.3 kg/m^2^ vs valid: 26.1 kg/m^2^), and were more likely to be smokers (not willing: 5.4% vs valid: 3.3%). Women who did not return their physical activity questionnaires, had a technical error with the accelerometer, or had insufficient wear time did not differ significantly in age (valid data: 70.0 years vs excluded: 69.9 years)), BMI (valid: 26.1 kg/m^2^ vs excluded: 26.6 kg/m^2^), or smoking (valid: 3.3% vs excluded: 3.5%) from the women included in the analytical sample.

The mean age of the 10,115 women in this analysis was 71.6 (SD = 5.7; range: 62.6, 100.7) years at the time of accelerometer assessment. Overall, women spent a median of 210.0 (63.5, 421.5) minutes per week in MVPA according to self-report. When assessed by accelerometer, the median minutes per week of MVPA was 512.0 (313.0, 742.0) using the vertical axis cutpoint of ≥760 cpm, 305.0 (168.0, 481.0) using ≥1041 cpm, 65.0 (21.0, 154.0) using ≥1952 cpm, and 142.0 (59.0, 270.0) using the vector magnitude ≥2690 cpm cutpoint.

**Fig 1:**
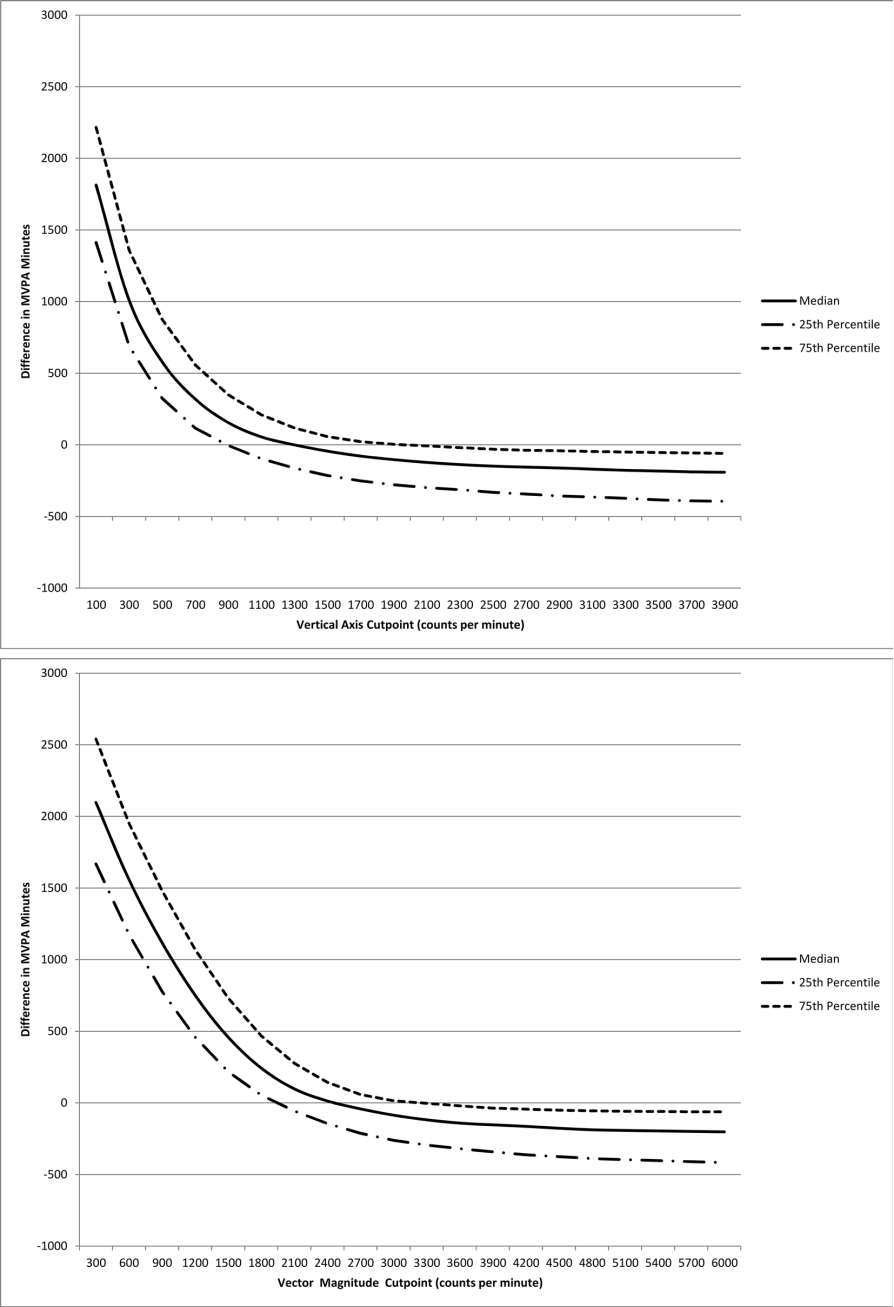
Difference Between Accelerometer-Assessed and Self-Reported Minutes per Week of Moderate-to-Vigorous Physical Activity by Cutpoint, Women’s Health Study, 2011–2014

The difference between the medians (interquartile range) of self-reported and accelerometer-assessed minutes per week of MVPA, using arbitrarily chosen vertical axis cutpoints ranging from ≥100 cpm to ≥3900 cpm was minimized when using the vertical cutpoint ≥1300 (900, 1900) cpm. Using vector magnitude cutpoints ranging from ≥300 cpm to ≥5900 cpm, the difference was minimized at the vector magnitude cutpoint ≥2400 (1950, 3300) cpm (Fig 1).

**Fig 2:**
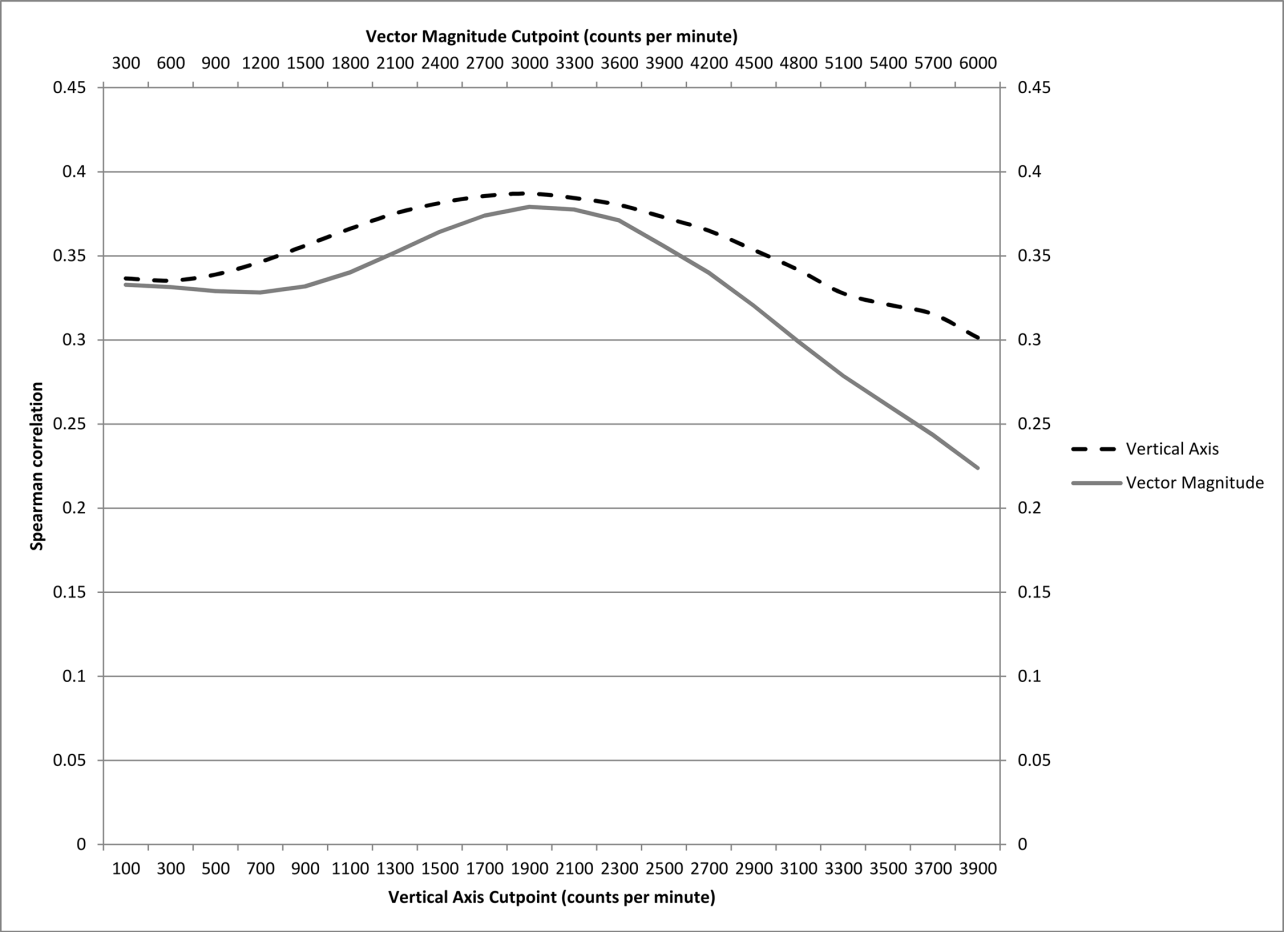
Correlation Between Accelerometer-Assessed and Self-Reported Moderate-to-Vigorous Physical Activity by Cutpoint, Women’s Health Study, 2011–2014.

Self-reported MVPA was moderately correlated with accelerometer-assessed MVPA. The 760 cpm cutpoint MVPA had a correlation of 0.35 (95% CI: 0.33, 0.37); 1041 cpm: ρ = 0.36 (95% CI: 0.35, 0.38), 1952 cpm: ρ = 0.39 (95% CI: 0.37, 0.40); and 2690 cpm: ρ = 0.37 (95% CI: 0.36, 0.39). When examining the correlation between self-reported MVPA minutes per week and accelerometer-assessed MVPA minutes per week using arbitrarily chosen vertical axis cutpoints ranging from ≥100 cpm to ≥3900 cpm, we observed correlations ranging from 0.30 to 0.39 with a maximum correlation at ≥1900 cpm (Fig 2). For triaxial accelerations, using vector magnitude cutpoints ranging from ≥300 cpm to ≥5900 cpm, the corresponding correlations ranged from 0.22 to 0.38 with a maximum correlation at ≥3000 cpm.

According to self-reported physical activity, 66.6% of women met the US federal physical activity guidelines, engaging in ≥150 minutes per week of moderate-intensity physical activity, ≥75 minutes of vigorous-intensity physical activity, or an equivalent combination (Table 1). The percent of women who met the guidelines according to uniaxial accelerometer-assessment decreased with greater required cpm for defining MVPA; ranging from 92.8% using ≥760 cpm to 26.0% using ≥1952 cpm. Using the triaxial cutpoint of ≥2690 cpm, 48.2% of women engaged in ≥150 minutes per week of MVPA. When requiring accelerometer-assessed activity to be performed in bouts ≥10 minutes, the proportion of women who met the guidelines decreased substantially across all accelerometer cutpoints (≥760 cpm: 50.0%, ≥1041 cpm: 33.0%, ≥1952 cpm: 13.4%, and ≥2690 cpm: 19.3%). There was poor classification agreement between self-report and accelerometer-assessment (Table 1). The strongest agreements between self-report and accelerometer-assessment was observed using the ≥760 cpm MVPA definition and requiring activity to be performed in bouts ≥10 minutes (kappa = 0.25), and the triaxial cutpoint of ≥2690 cpm without the minimum bout length restriction (kappa = 0.25).

**Table 1:** Percent of Women who Meet US Federal Physical Activity Guidelines by Self-Report and Accelerometer-Assessment, Women's Health Study, 2011–2014.

Self-Reported		Accelerometer-Assessed: No Minimum Bout Length^c^											
		Vertical Axis: 760 cpm			Vertical Axis: 1041 cpm			Vertical Axis: 1952 cpm			Vector Magnitude: 2690 cpm		
		Meeting guidelines^b^	Not meeting guidelines	Kappa	Meeting guidelines	Not meeting guidelines	Kappa	Meeting guidelines	Not meeting guidelines	Kappa	Meeting guidelines	Not meeting guidelines	Kappa
Meeting guidelines^a^	66.6%	63.5%	3.1%	0.09	56.4%	10.2%	0.21	22.6%	44.1%	0.18	38.4%	28.2%	0.25
Not meeting guidelines	33.4%	29.3%	4.1%		21.9%	11.4%		3.4%	29.9%		9.8%	23.6%	
		92.8%	7.2%		78.3%	21.7%		26.0%	74.0%		48.2%	51.9%	
Self-Reported		Accelerometer-Assessed: 10-Minute Minimum Bout Length^d^											
		Vertical Axis: 760 cpm			Vertical Axis: 1041 cpm			Vertical Axis: 1952 cpm			Vector Magnitude: 2690 cpm		
		Meeting guidelines^b^	Not meeting guidelines	Kappa	Meeting guidelines	Not meeting guidelines	Kappa	Meeting guidelines	Not meeting guidelines	Kappa	Meeting guidelines	Not meeting guidelines	Kappa
Meeting guidelines^a^	66.6%	39.6%	27.0%	0.25	28.2%	38.5%	0.22	12.4%	54.3%	0.11	17.5%	49.1%	0.15
Not meeting guidelines	33.4%	10.4%	23.0%		4.8%	28.6%		1.0%	32.4%		1.8%	31.6%	
		50.0%	50.0%		33.0%	67.1%		13.4%	86.6%		19.3%	80.7%	

Abbreviations: CPM, counts per minute.

^a^Meeting guidelines is defined as ≥150 minutes per week of moderate-to-vigorous physical activity (MVPA), where time is calculated as a sum of time over all moderate- and vigorous-intensity activities (≥3.0 METs); not meeting, otherwise.

^b^Meeting guidelines is defined as ≥150 minutes per week where the accelerometer registers at least 760 cpm [31], 1041 cpm [30], 1952 cpm [28], or 2690 cpm [39], equivalent to MVPA; not meeting, otherwise.

^c^Not restricted to a minimum bout length.

^d^Bouts of at least 10 minutes.

## Discussion

It is necessary to understand how the use of different cutpoints affects MVPA estimates, as well as how these estimates relate to self-reported physical activity in order to compare results across different studies.[46, 47] There does not appear to be an appropriate or even an overwhelmingly preferred accelerometer cutpoint to define MVPA in older adults, resulting in recent studies having used different cutpoints to define MVPA.[27, 31–33, 48–50] In a large sample of older women, we examined the differences in MVPA estimates from self-report and accelerometer-assessment across four commonly employed MVPA cutpoint definitions. In addition, this study provides one of the first descriptions of MVPA according to triaxial accelerometer assessment in older women.

### Accelerometer-assessed MVPA across varying cutpoints

We defined accelerometer-assessed MVPA using four cutpoints previously proposed in the literature.[28, 30, 31, 39] Matthews et al developed the ≥760 cpm cutpoint by aggregating information across several laboratory and field studies to determine a cutpoint that would reliably capture time spent at 3.6 METs or greater.[31] Copeland et al developed the ≥1041 cpm cutpoint in a sample of 38 older adults (mean age 70 years) in a laboratory-based calibration study.[30] Freedson et al developed the ≥1952 cpm cutpoint in a sample of 50 adults, mean age 24 years, from treadmill protocols using indirect calorimetry as the measure of energy expenditure.[28] Similarly, Sasaki et al developed the triaxial cutpoint of ≥2690 cpm using the vector magnitude of counts from all three axes in a sample of 36 adults from treadmill protocols using an identical protocol as the Freedson et al study.[39]

The WHS data showed that accelerometer-assessed MVPA differed by cutpoint and from self-reported assessment. WHS women spent a median of 210 minutes per week in MVPA based on self-report. In contrast, accelerometer estimates ranged from 65–512 minutes per week depending on the MVPA cutpoint employed. The triaxial MVPA cutpoint provided the most similar median and lower interquartile range estimate of accelerometer-assessed MVPA when compared to the self-reported MVPA.

Evenson et al described how estimates of time in MVPA differed across arbitrary cutpoints in a nationally representative sample of 1,314 US adult women aged 60 years and older. Similarly to WHS, albeit with different cutpoints, a definition requiring greater cpm decreased the minutes spent in MVPA ranging from a mean of 699 minutes per week using a ≥500 cpm cutpoint to 60.9 minutes per week using a ≥2000 cpm cutpoint.[27] Hamer et al observed, in a sample of 394 civil service adults (mean age: 54 years), that adults spent an average of 261 minutes per week in MVPA, using a ≥2000 cpm definition of MVPA.[51] Hagstromer et al, in a national survey among 263 US women aged 60 to 75 years, reported a mean of 430 minutes per week between 760 cpm and 2019 cpm and 70 minutes per week using the ≥2020 cpm cutpoint.[52] However, there are no comparable data using the triaxial cutpoint of ≥2690 cpm in older women. These data suggest WHS women are similar to or slightly more active than the other populations, including several nationally representative samples.

### Comparing self-reported and accelerometer-assessed MVPA

Considerable research has demonstrated that self-report physical activity questionnaires are reliable, but only moderately correlated with objective measures.[12, 13, 18, 41, 53, 54] The current study correlations of 0.35 to 0.39 between self-report and accelerometer-assessed MVPA are similar to those observed in other studies, which use the three cutpoints.[14, 30, 48, 53, 55, 56] For example, Pettee Gabriel reported a correlation between accelerometer-assessment, using ≥1952 cpm cutpoint, and the Nurses Health Study II questionnaire (a similar questionnaire to the one used in the current study) of 0.38.[14] Dyrstad et al note a correlation of 0.27 when comparing MVPA at the ≥760 cpm threshold and self-reported MVPA using the International Physical Activity Questionnaire (IPAQ).[20] Van Hoole et al, also using the IPAQ, observed a correlation of 0.33 to 0.40 for total weekly minutes of MVPA, when using the ≥1952 cpm cutpoint, among a group of older adults.[18]

Use of triaxial data does not appear to substantially improve the correlation between self-report and accelerometer assessment, compared to uniaxial data, however the median and 25^th^ percentile of MVPA minutes were closest to self-reported assessment when using the vector magnitude cutpoint. We examined the correlation between minutes per week of MVPA by self-report and accelerometer assessment across a range of arbitrary cutpoints for both uniaxial (≥100 cpm to ≥3900 cpm) and triaxial data (≥300 cpm to ≥5900 cpm). The maximum correlation for both uniaxial and triaxial data was approximately 0.38. For uniaxial data, this maximum occurred at ≥1900 cpm, while the triaxial maximum was at ≥3000 cpm. The uniaxial cutpoint that minimized the difference between self-reported and accelerometer-assessed minutes per week of MVPA was ≥1300 cpm; the triaxial cutpoint, ≥2400 cpm.

According to self-reported physical activity, 67% of women met the US federal physical activity guidelines, engaging in ≥150 minutes per week of MVPA. The percent of women who met the guidelines varied widely depending on the accelerometer MVPA definition (≥760 cpm: 50%, ≥1041 cpm: 33%, ≥1952 cpm: 13%, and ≥2690 cpm: 19%). In a national survey of 1018 individuals aged 60 years and older, Tucker et al compared self-reported physical activity with accelerometer-assessment using ≥2020 cpm as the MVPA cutpoint.[24] A similar proportion of WHS women and adults in the national sample met the guidelines by self-report (60%), but a slightly larger portion of WHS women met the guidelines by accelerometry (13% vs. 8.5% in those 60–69 years and 6.3% in those ≥70 years).

### MVPA assessment challenges and innovations

Potentially the largest challenge in comparing data collected using accelerometers or questionnaires is that they measure related but not identical constructs: accelerometers measure accelerations in physical motion, and do not directly measure behavior.[16] Furthermore, where participants wear the accelerometer may affect physical activity estimates. Wearing the accelerometer on the hip will not assess well activities that employ primarily the upper body or have limited hip movement such as stationary cycling or weight lifting, particularly if uniaxial devices are employed. Because of this, several researchers have called into question the appropriateness of directly comparing accelerometer-assessed and self-reported physical activity.[16, 57] However, with the increased use of accelerometers due to objectivity, ease of wear, and 24-hour monitoring, it remains necessary to understand and quantify the differences in physical activity estimates from these two assessment methods. In this study, we not only examined the correlation between the two measures, but also the absolute difference in estimates. While accelerometers offer the possibility of more detailed characterization of physical activity (e.g., identification of short bouts), innovative analytical methods such as pattern analysis, machine-learning, and the sedentary sphere which may allow better identification of behavior (rather than movement) are still under development.[34, 35] The standard and currently accepted method for translating accelerometer data is the use of simple regression analyses which calibrate energy expenditure performing activities of different intensities (e.g., using indirect calorimetry) against counts obtained from the accelerometer.[28, 32, 58] However, available calibration studies have used different protocols and are generally conducted in small samples of young and healthy populations.[28, 31] It is unclear if cutpoints obtained under these conditions are universally appropriate for all populations.

The cutpoints used in the present study have been shown in laboratory settings, mainly using treadmill walking protocols, to reliably and consistently estimate moderate-intensity activity.[29, 59] However, few studies have specifically examined older populations in a free-living environment and included non-walking activities, particularly household activities and light-intensity activities. This may account for the lack of consensus on an appropriate cutpoint for older adults. In a sample of adults aged 60 years and older, Hall et al. showed that although walking on a treadmill and walking in a hallway at the same speed generated the same energy expenditure, they resulted in different mean accelerometer counts per minute,[33] indicating that laboratory-based cutpoints may not translate well when applied to similar activities carried out in the real world.

Current physical activity guidelines were developed using data from studies of using self-reported activity, and not device-assessed activity, in relation to disease development.[3, 8, 16] However, several ongoing studies [60–63] currently are investigating accelerometer-assessed physical activity in relation to disease outcomes and will provide results in the near future, informing future guidelines. However, while self-report and accelerometer-assessed physical activity do not measure identical constructs, examining how participants are classified or ranked, using the metric of 150 minutes per week, may be useful.

### Strengths & limitations

The main strength of this study is a large sample of >10 000 older women, in whom we simultaneously examined assessments from self-report and accelerometer across a range of cutpoints. In particular, this study is one of the first to describe physical activity among older women using triaxial data. However, we did not have a “gold-standard” assessment of MVPA to directly compare self-reported and accelerometer-assessed MVPA. In addition, we did not ask women to limit their reporting of physical activity only to those lasting at least 10 minutes, as required by current guidelines. Furthermore, these data are also limited to WHS participants, who are older women, mostly white, and of higher socioeconomic status; however, comparison of their data to national samples indicate similar levels of physical activity.[64]

## Conclusion

We show that the choice of accelerometer cutpoint impacts MVPA estimation. Among the cutpoints examined, the triaxial accelerometer MVPA cutpoint compared to self-report yields the most similar median, and lowest interquartile range of MVPA minutes per week. However, use of uniaxial and triaxial cutpoints yielded similar correlations when compared with self-reported physical activity. Although cutpoints may be a simplistic use of the rich accelerometer data, this is the only well-studied metric available today, pending further development of methods.

## Supporting Information

S1 Data Women’s Health Study: De-identified dataset. (ZIP)

S1 Fig Women’s Health Study: Accelerometer Ancillary Study Physical Activity Questionnaire, 2011–2014. (TIF)

## Abbreviations

CI: confidence interval
CPM: counts per minute
MET: metabolic equivalent
MVPA: moderate-to-vigorous physical activity
WHS: Women’s Health Study
