# Supplementary figures and images for: Comparison of Self-Reported and Accelerometer-Assessed Physical Activity in Older Women
Source: PLoS One. 2015 Dec 29;10(12):e0145950. doi: 10.1371/journal.pone.0145950 (PMC4694656; doi:10.1371/journal.pone.0145950)

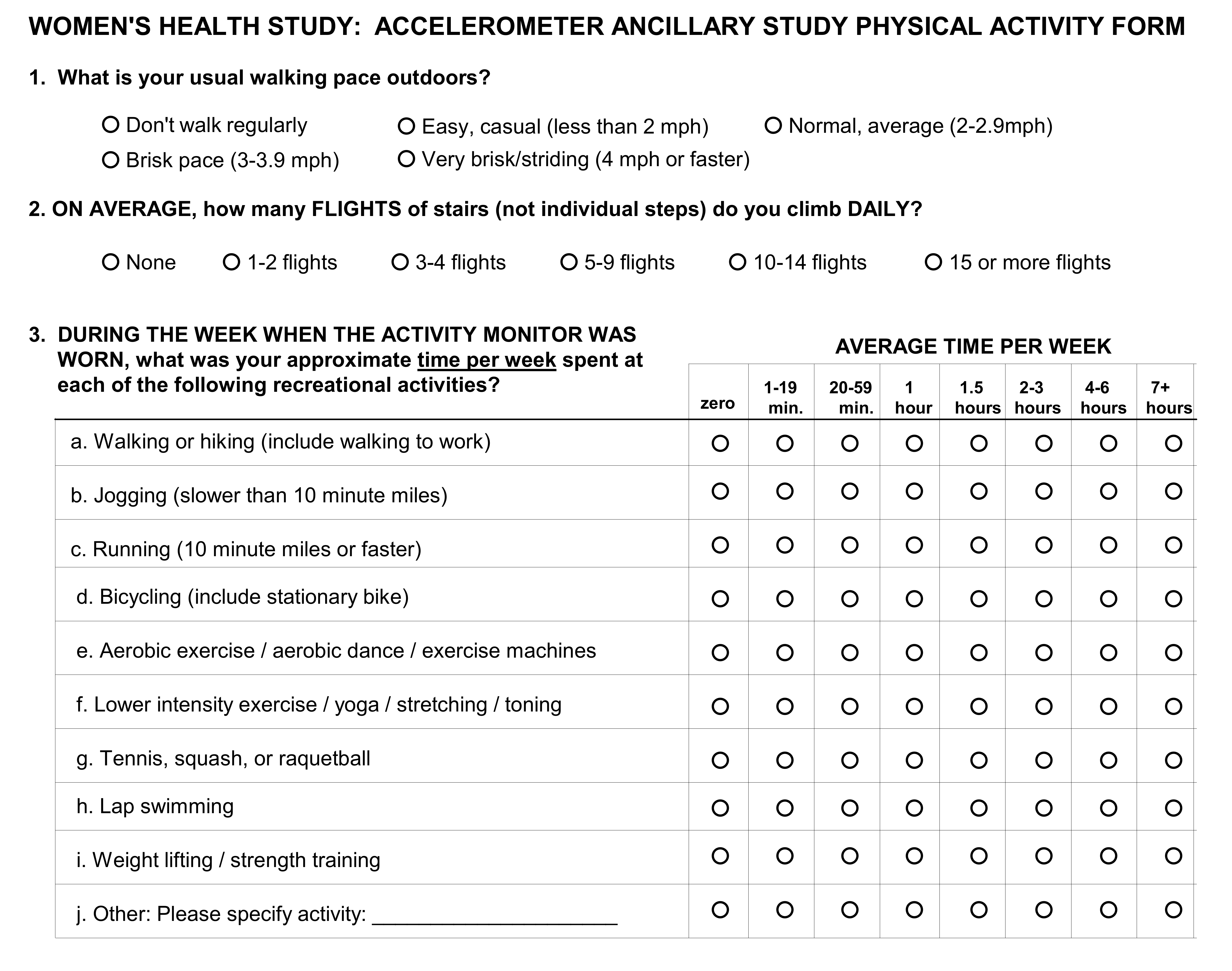

Supplement: S1 Fig — (TIF) [file pone.0145950.s002.tif]
